# An anti-diabetic drug targets NEET (CISD) proteins through destabilization of their [2Fe-2S] clusters

**DOI:** 10.1038/s42003-022-03393-x

**Published:** 2022-05-10

**Authors:** Henri-Baptiste Marjault, Ola Karmi, Ke Zuo, Dorit Michaeli, Yael Eisenberg-Domovich, Giulia Rossetti, Benoit de Chassey, Jacky Vonderscher, Ioav Cabantchik, Paolo Carloni, Ron Mittler, Oded Livnah, Eric Meldrum, Rachel Nechushtai

**Affiliations:** 1 The Alexander Silberman Institute of Life Science and The Wolfson Centre for Applied Structural Biology, Faculty of Science and Mathematics, The Edmond J. Safra Campus at Givat Ram, The Hebrew University of Jerusalem, Jerusalem, 91904 Israel; 2 Department of Physics, RWTH Aachen University, 52074 Aachen, Germany; 3 Department of Surgery, University of Missouri School of Medicine, and Interdisciplinary Plant Group, Christopher S. Bond Life Sciences Center, University of Missouri, 1201 Rollins St, Columbia, MO 65211 USA; 4 Computational Biomedicine Section, Institute of Advanced Simulation IAS-5 and Institute of Neuroscience and Medicine INM-9, Forschungszentrum Jülich GmbH, 52425 Jülich, Germany; 5 Computational Biomedicine, Institute of Advanced Simulation IAS-5 and Institute of Neuroscience and Medicine INM-9, For-schungszentrum Jülich GmbH, 52425 Jülich, Germany; 6 ENYO‐Pharma, Bioserra 1, 60 Avenue Rockefeller Bâtiment B, 69008 Lyon, France; 7 JARA Institute: Molecular Neuroscience and Imaging, Institute of Neuroscience and Medicine INM-11, Forschungszentrum Jülich GmbH, 52425 Jülich, Germany

**Keywords:** Molecular medicine, Mechanisms of disease

## Abstract

Elevated levels of mitochondrial iron and reactive oxygen species (ROS) accompany the progression of diabetes, negatively impacting insulin production and secretion from pancreatic cells. In search for a tool to reduce mitochondrial iron and ROS levels, we arrived at a molecule that destabilizes the [2Fe-2S] clusters of NEET proteins (M1). Treatment of db/db diabetic mice with M1 improved hyperglycemia, without the weight gain observed with alternative treatments such as rosiglitazone. The molecular interactions of M1 with the NEET proteins mNT and NAF-1 were determined by X-crystallography. The possibility of controlling diabetes by molecules that destabilize the [2Fe–2S] clusters of NEET proteins, thereby reducing iron-mediated oxidative stress, opens a new route for managing metabolic aberration such as in diabetes.

## Abstract (web summary)

Marjault et al. report two crystal structures of a drug molecule bound to NEET proteins mNT and NAF-1 and the effects of this molecule on diabetic mice, suggesting an approach for reducing mitochondrial iron and ROS levels in diabetic patients.

## Introduction

NEET proteins are a relatively new class of Fe-S protein. In humans, NEET proteins are represented by MiNT, localized inside the mitochondria, mitoNEET, and NAF-1, both anchored to the mitochondria outer membrane; with NAF-1 being also present on the surface of the endoplasmic reticulum and its mitochondria-associated membrane^1^. NEET proteins harbor two [2Fe-2S] clusters with a unique 3Cys:1His coordination structure^2^. These display unique lability characteristics that are dependent on their oxidative state and immediate environment^1,2^. NEET proteins were found to be involved in mitochondrial Fe/Fe-S metabolism oxidative stress management^3^. Indeed, a growing amount of evidence demonstrate that NEET proteins play a significant role in Fe/Fe-S exchange between mitochondria and cytosol in cells^3–5^. In addition, NEET proteins and their functions were associated with numerous diseases, ranging from neurodegeneration to metabolic diseases, such as diabetes^1^. In humans, the complete loss of NAF-1 due to mutations in the *CISD2* gene, causes Wolfram Syndrome 2^6–9^, a progressive neurodegenerative disorder characterized by optic atrophy, sensory hearing loss, defective platelet aggregation and severe insulin deficiency, leading to juvenile-onset of diabetes mellitus^6,10,11^. Pioglitazone and other molecules of the TDZ family were shown to bind and stabilize the [2Fe-2S] clusters of NEET proteins^4,12,13^. The stabilization of the [2Fe-2S] clusters of NEET protein in cells suppresses the Fe/Fe-S exchange between the mitochondria and the cytosol, inhibiting the function of NEET protein in mitochondrial Fe/Fe-S export^4^. In diseases such as diabetes (associated with or without obesity), the expression of NEET proteins is decreased, potentially causing an acute mitochondrial iron accumulation and oxidative stress^14–16^. High levels of mitochondrial oxidative stress were also linked with acute mitochondrial iron accumulation during diabetes^17–20^. Relief of mitochondrial oxidative stress was therefore proposed as a therapeutic approach to treat metabolic diseases, and to date, only a few solutions are available^17,21^. Here, we propose to take advantage of the function of NEET proteins and the lability of their [2Fe-2S] clusters. In contrast to stabilization of NEET [2Fe-2S] clusters, which suppresses mitochondrial Fe/Fe-S export and cause a buildup of iron and ROS inside the mitochondria, we propose in acute situations to relieve mitochondrial oxidative stress by increasing the lability of the [2Fe-2S] clusters of the NEET proteins (that would facilitate [2Fe-2S] cluster transfer from the mitochondria to the cytosol, decreasing mitochondrial iron and ROS content). To achieve that, we identified the novel molecule, M1, that enhances the lability of the [2Fe-2S] clusters of mNT and NAF-1 protein and demonstrated its potential to lower mitochondrial iron and ROS accumulation and successfully treat diabetic mice.

## Results

### M1 enhances the lability of the [2Fe–2S] clusters of mNT and NAF-1

**Fig. 1:**
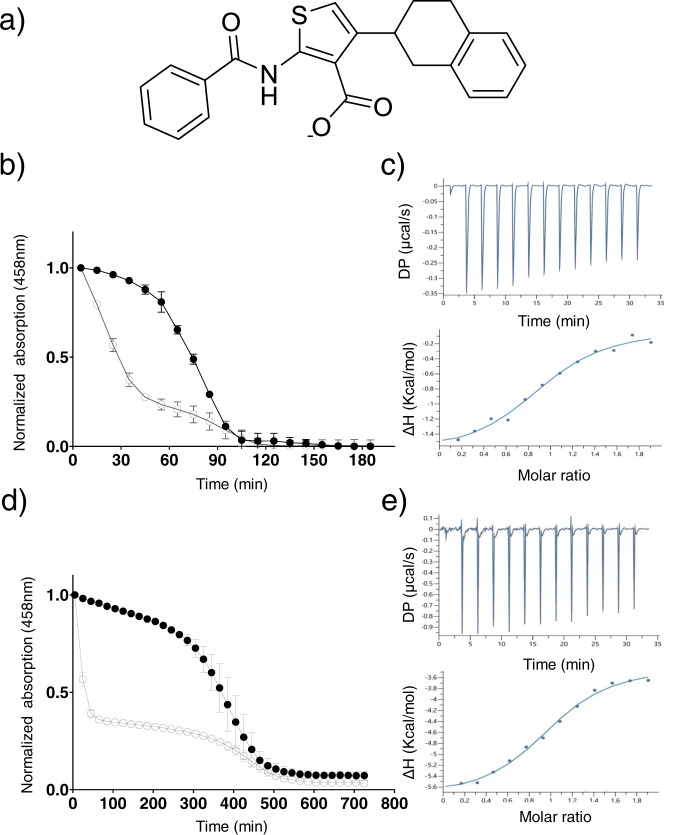
M1 enhances the lability of mNT and NAF-1’s [2Fe–2S] clusters. **a** Structure of the M1 molecule. The [2Fe-2S] cluster-release from 20 µM of mNT (**b**) or 20 µM NAF-1 (**d**) NEET proteins was monitored by UV-Vis absorption spectroscopy. The 458 nm characteristic absorption peak of [2Fe–2S] clusters of the NEET proteins which were incubated with- (empty circle) or without (filled circle) 60 µM of M1 molecule, at a temperature of 37 °C and pH 6.0. Error bar represents the standard deviation of three independent experiments. ITC binding curve of M1 to mNT (**c**) or to NAF-1, (**e**). 50 µM of proteins (mNT or NAF-1) were titrated against 500 µM of M1 by injecting 3 µL for 13 injections. Each measurement was repeated in three independent experiments.

The [2Fe–2S] clusters of NEET proteins are labile in a manner that is dependent on their oxidation state and microenvironment pH. Under reduced conditions or basic pH, the reduced [2Fe–2S] clusters are stably bound to NEET proteins^22–24^. However, under low pH, or oxidized conditions, the [2Fe–2S] clusters of NEET proteins dissociate quickly^1,24,25^. To date, several small molecules capable of stabilizing the labile NEET protein’s [2Fe–2S] clusters have been reported^13,26^. Here we report, for the first time, on a chemical compound, M1 (Fig. 1a), that accelerates the dissociation of the [2Fe–2S] clusters of mNT and NAF-1 in a dose dependent manner (Supplementary Fig. 1). The new compound, named M1, was discovered following the same method used to identify the new compound Mito-C, a stabilizer of NAF-1’s [2Fe-2S] clusters^27,28^. Using the specific UV-VIS absorption peak of NEET protein bound [2Fe–2S] clusters at 458 nm^29^, the rate of dissociation of mNT or NAF-1 [2Fe–2S] clusters were measured in the presence/absence of the M1 molecule. When incubated with the M1 molecule, the dissociation of mNT or NAF-1 [2Fe–2S] clusters were accelerated by more than two-fold. The time to achieve 50% loss was reduced by M1 from 80 min (untreated control) to 30 min for mNT (Fig. 1b) and from 340 min to 30 min for NAF-1 (Fig. 1d). Using isothermal titration calorimetry (ITC), the dissociation constant of M1-mNT was measured to be 5.93 µM (±0.53) (Fig. 1c) and 7.2 µM (±0.99) for NAF-1 (Fig. 1e). These measurements were carried out in acidic pH to ensure that the proteins’ dynamics and cluster destabilization is minimal^4,10^.

### Increased lability of NEET protein [2Fe–2S] clusters by M1 relieves mitochondrial iron and ROS accumulations in INS-1E cells with suppressed mNT or NAF-1 expression

**Fig. 2:**
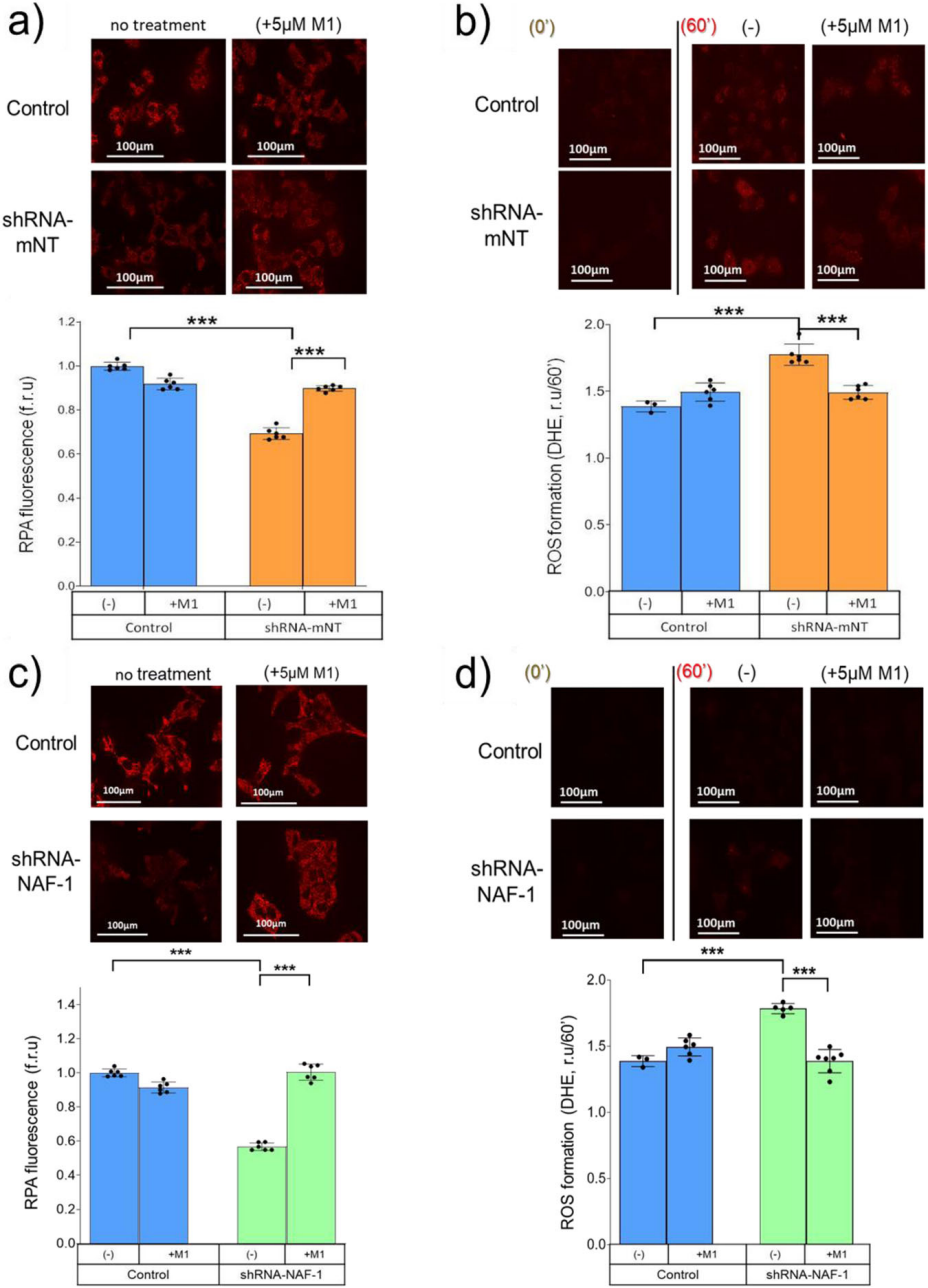
M1 alleviates mitochondrial iron and ROS accumulation caused by reduced expression of mNT or NAF-1 in INS-1E cells. mLI levels of normal (control, INS-1E cells transfected with empty vector - blue bars) and INS-1 E cells with suppressed expression of mNT [shRNA-mNT (**a**. orange bars)] or NAF-1 [shRNA-NAF-1 (**c** green bars)] were assessed by the (rhodamine B-[(1,10-phenanthrolin-5-yl aminocarbonyl] benzyl ester) RPA probe. Upper panels: Semi-Confocal microscopic images of RPA fluorescence in the mitochondria of control and INS-1E cells with reduced expression of mNT or NAF-1 following pretreatment of cells with or without 5 µM of M1 for 30 min. Decreased RPA fluorescence indicates increased mitochondrial labile iron. Lower panels: Quantitative analysis of RPA fluorescence relative to untreated control cells following pretreatment of cells with or without 5 µM M1 for 30 min prior to RPA addition. Mitochondrial ROS accumulation determined with the mitochondrial specific mito-SOX^TM^ probe. Upper panels: Epi-fluorescence images of control (transfected with empty vector) and INS-1E cells with reduced mNT (**b**) or NAF-1 (**d**) expression following pre-treatment of cells with or without 5 µM of M1 for 30 min. After the pre-treatment, ROS formation was detected over 60 min using mito-SOX^TM^. Lower panel: Quantitative analysis of mito-SOX^TM^ fluorescence change over 60 min incubation for treated or not treated cells with normal mNT protein expression (control/blue) and suppressed mNT expression cell (shRNA-mNT/orange) (**b**) or NAF-1 (shRNA-NAF-1/green) (**d**). Analysis was performed with (image J). ***Indicates *P* < 0.001, *n* = 6.

NEET proteins play a crucial role in the maintenance of iron and ROS homeostasis in cells^3^ with increases in the mitochondrial labile iron (mLI) pool known to induce accumulation of mitochondrial reactive oxygen species (mROS)^30,31^. The resulting mitochondrial stress leads to mitochondrial fragmentation^32^, a decrease in ATP production^33,34^, and cell death^30,31^. Consistent with this, reduced expression of either mNT or NAF-1 using shRNA, induces mLI and mROS accumulation in numerous cell types^5,35,36^. Moreover, in vivo studies in *ob/ob* diabetic mice with lowered mNT expression demonstrate significant accumulation of mLI and mROS^15^. Here, we show that M1-induced lability of NEET protein [2Fe–2S] clusters repairs the mLI and mROS accumulation that results from decreased expression of mNT or NAF-1 in INS-1E *β*-cells. INS-1E *β*-cells were transfected with shRNA directed against mNT or NAF-1 mRNA, leading to an approximately 50% decrease in mNT or NAF-1 expression (Supplementary Fig. 2). Using the mitochondrial iron-fluorescent sensor rhodamine B-[(1,10-phenanthroline-5-yl aminocarbonyl] benzyl-ester (RPA), accumulation of mitochondrial iron was detected in INS-1E *β*-cells with lowered mNT (Fig. 2a) or NAF-1 (Fig. 2c) expression. As a consequence of the higher mLI, mROS also accumulates to higher levels in INS-1E *β*-cells with lowered mNT (Fig. 2b) or NAF-1 (Fig. 2d) expression. Figure 2 shows that mitochondrial iron and ROS accumulation, resulting from lowered mNT or NAF-1 expression, can be relieved by treatment with the M1 molecule. We hypothesize that this is a consequence of enhanced export of mitochondrial Fe-S clusters as a result of accelerating the release of [2Fe–2S] clusters from the remaining mNT proteins, or NAF-1, induced by M1. Moreover, in a previous study, it was shown that mNT protein expression did not increasing when the expression of NAF-1 was downregulated^37^. Here, in a similar experiment, we showed that NAF-1 expression is not increasing when mNT protein expression is downregulated (Supplementary Fig. 3).

### M1 reverses the decreased insulin content and secretion in INS-1E *β*-cells with suppressed

#### mNT or NAF-1 expression

**Fig. 3:**
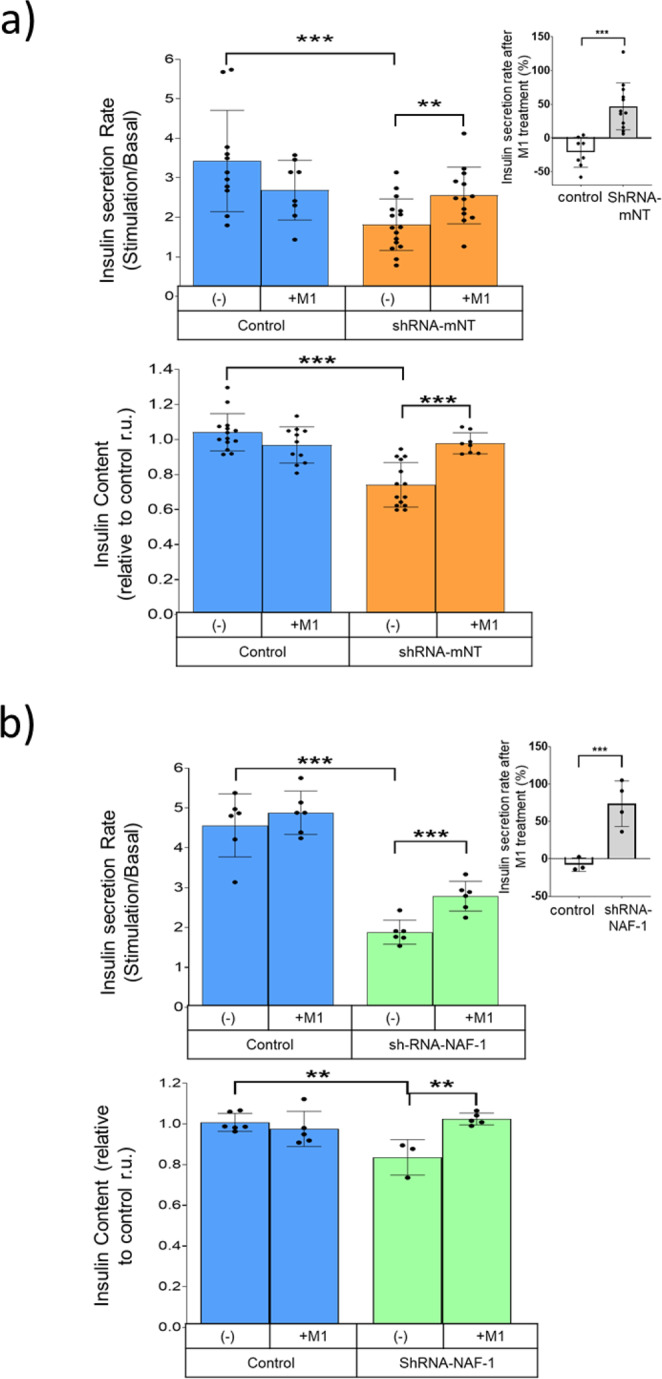
M1 restores the decreased insulin content and secretion of INS-1E *β*-cells with suppressed expression of mNT and NAF-1 NEET proteins. **a** (Upper) Insulin secretion rate of INS-1E cells with suppressed mNT expression (shRNA-mNT/orange) and cells expressing wild type levels of mNT (blue) following treatment with or without 5 µM of M1. Upper right graph shows the percentage of insulin secretion rate change from the different cells indicated after M1 molecule treatment. (Lower) INS-1E cell Insulin content measurements relative to untreated control following treatment with or without 5 µM M1. **P < 0.01, ***P < 0.001. n = 6. **b** (Upper) Insulin secretion rate of INS-1E cells with suppressed NAF-1 expression (shRNA-NAF-1/green) and cells expressing normal levels of NAF-1 (blue) following treatment with or without 5 µM of M1. Upper right graph shows the percentage of insulin secretion rate change from the different cells indicated after M1 treatment. (Lower) INS-1E cell Insulin content measurements relative to control levels following treatment with or without 5 µM of M1. **P < 0.01, ***P < 0.001. n = 6.

Since their discovery as targets of the anti-diabetes drug pioglitazone, NEET proteins have been implicated in diabetes associated cell pathophysiology^1,26^. INS-1E *β*-cells with decreased expression levels of mNT or NAF-1 showed a significant decrease in insulin secretion rate upon glucose stimulation compared to control cells (Fig. 3a, b. upper panels). Furthermore, suppressed expression of mNT or NAF-1 in INS-1E *β*-cells resulted in significantly diminished insulin content with a higher effect in the shRNA-mNT cell line (Fig. 3a, b, lower panels). In both cell lines with suppressed NEET protein expression, 3 h of exposure to M1, corrected the impaired glucose-stimulated insulin secretion rate and resulted in an almost complete correction of cellular insulin content, reflecting a restoration of insulin synthesis (Fig. 3a, b).

#### M1 restores glucose sensitivity in *db/db* mice without inducing weight gain

**Fig. 4:**
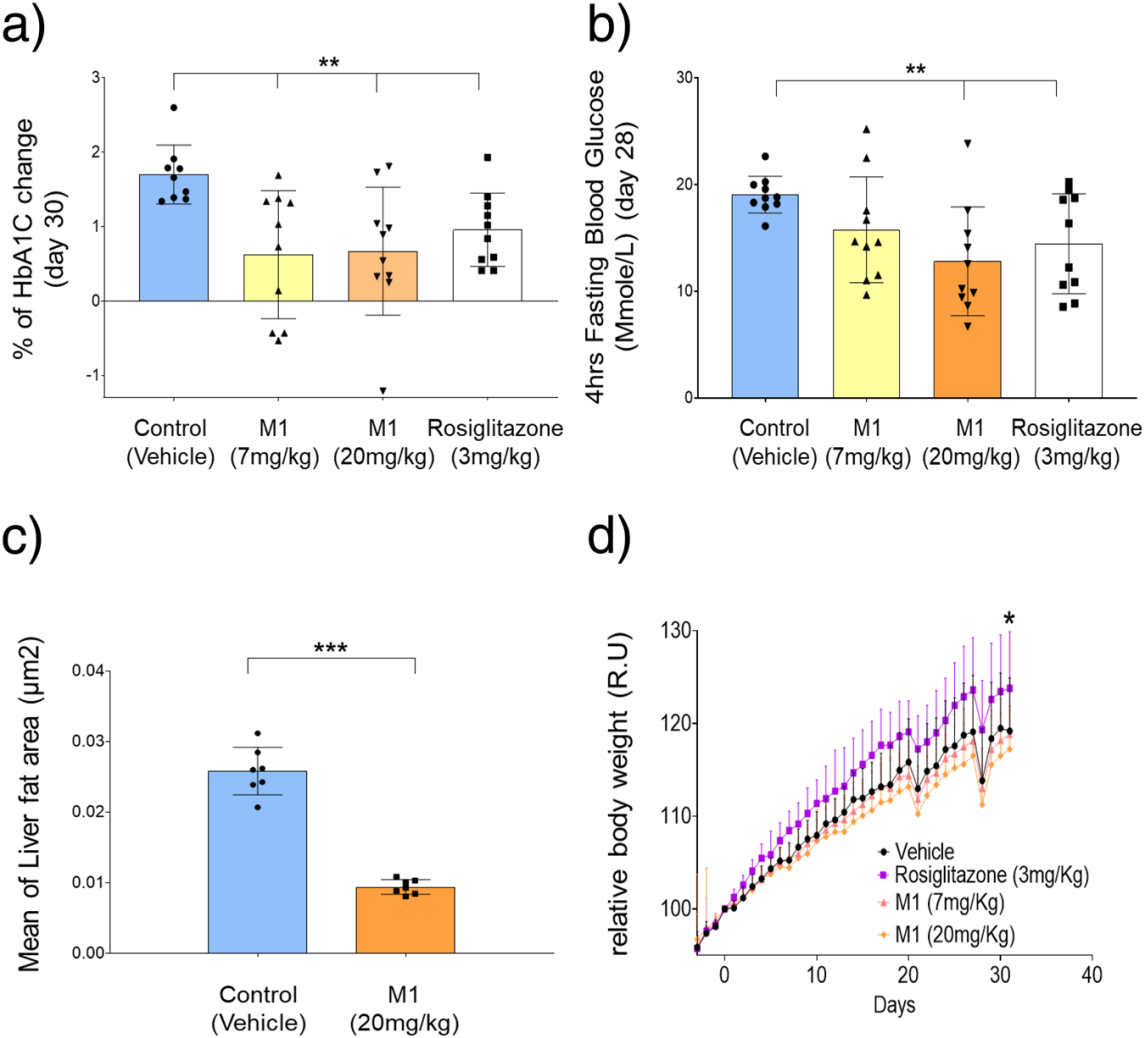
Oral administration of M1 ameliorates glycemic parameters in *db/db* mice. **a** Percentage change (relative to predosed randomization measurements on day-3) in circulating HbA1c after 31 days treatment with 7 mg/kg (yellow) or 20 mg/kg (orange) M1 and 3 mg/kg of rosiglitazone (white). **b** Blood glucose levels after a 4 h fast following 28 days of treatment with 7 mg/kg (yellow) or 20 mg/kg (orange) M1 and 3 mg/kg (white) of rosiglitazone. **c** Mean liver fat area following histological assessment after 31 days treatment with 20 mg/kg (orange) **d** Body weight as a percentage of day 0 following treatment of mice with 7 mg/kg (red circle) and 20 mg/kg (orange diamond) M1, 3 mg/kg rosiglitazone (purple circle) and vehicle control (black circle). *<0.05, **P < 0.01, ***P < 0.0001. *n* = 10.

To determine if oral administration of M1 and its effects on NEET proteins could ameliorate diabetic phenotypes in mice, we examined the impact of M1 on glycemic parameters in *db/db* mice. For 31 days, treatment groups were orally administered with 7 mg/kg or 20 mg/kg M1, vehicle alone, or 3 mg/kg of the insulin sensitizing drug rosiglitazone, for comparison. Over the course of the study, M1 treatment resulted in a significant slowing, relative to vehicle treated control, of time dependent blood glucose increase as assessed by glycated hemoglobin (HbA1c) (Fig. 4a). Furthermore, a significant improvement in blood glucose levels after a 4 h fast on day 28 of treatment (Fig. 4b) was observed as a consequence of M1 treatment. Both of these effects indicate that M1 molecule administration improved glucose control in *db/db* mice. The M1 molecule, did not affect circulating insulin, triglycerides, or total cholesterol. Interestingly, histopathological inspection of hepatic fat accumulation demonstrated a significant reduction of hepatic lipids in response to M1 molecule administration (Fig. 4c). Finally, no significant effect of the M1 molecule was observed on weight gain during the course of the study relative to vehicle treated control (Fig. 4d). This finding was in contrast to the rosiglitazone treatment which accentuated the rate of weight gain over the study (Fig. 4d; an established class effect of TZDs reported previously in preclinical and clinical settings).

#### The binding of M1 to mNT and NAF-1 revealed by X-ray crystallographic analysis

**Fig. 5:**
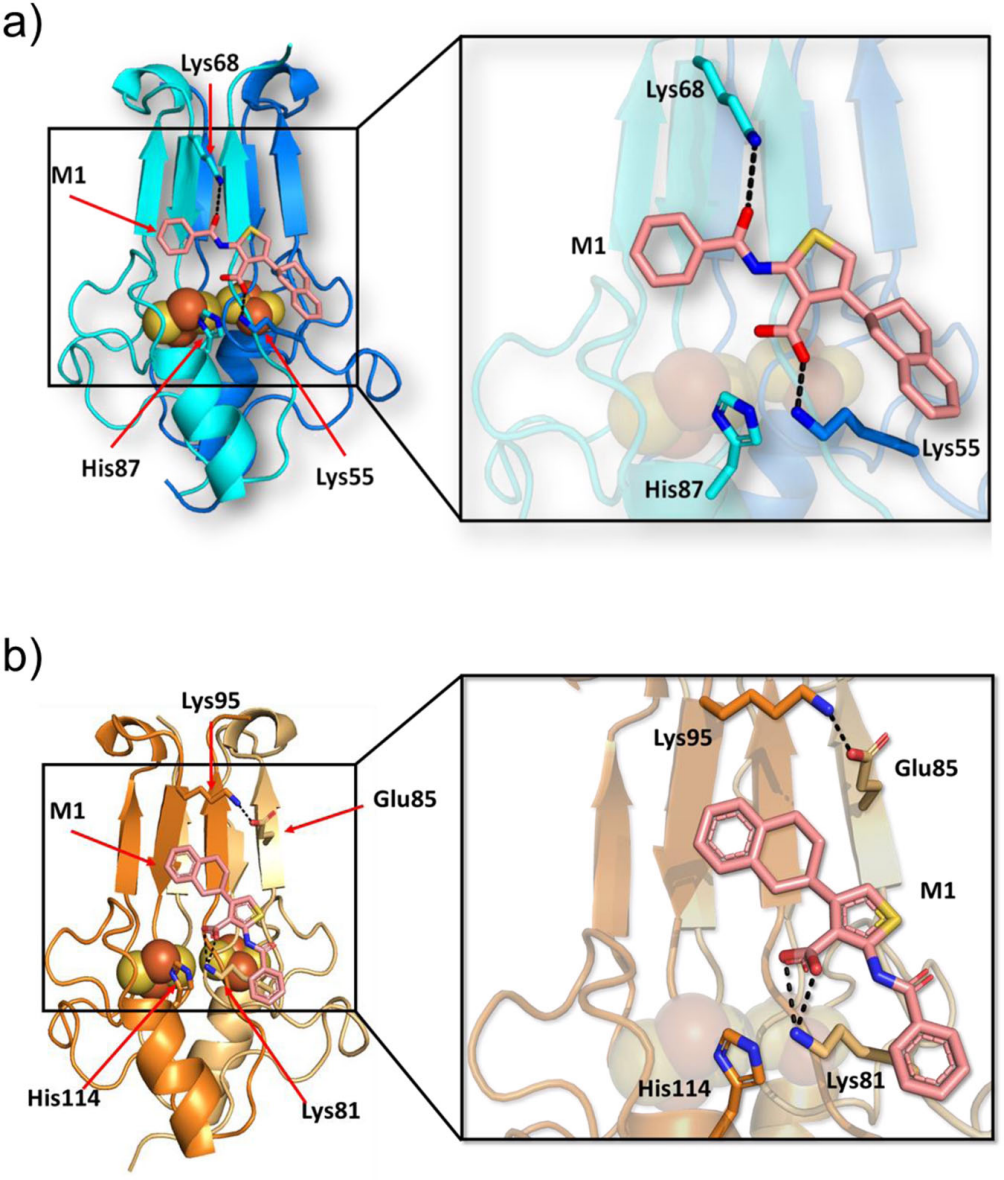
Structure of M1-mNT and M1-NAF-1. **a** M1-mNT co-crystals were obtained at pH 8.0 by co-crystallization. The crystals had X-ray diffraction to 1.64 Å resolution (Supplementary Table 1 and PDB code: 7P0O). The two monomer subunits of mNT are colored in cyan and marine while M1 is represented in stick format. M1 forms hydrogen bonds (black dash) with the side chain of Lys68 via its amide oxygen atom, and a salt bridge (black dash) with Lys55 via its carboxyl oxygen atom (box). **b** M1-NAF-1 co-crystals were obtained at pH 8.0 by co-crystallization, with crystals showing X-ray diffraction to 1.74 Å resolution (Supplementary Table 1 and PDB code: 7P0P). The two monomer subunits of NAF-1 are colored in Orange and light orange while M1 is represented in stick format. M1 forms hydrogen bonds (black dash) with the side chain of Lys 81 via its carboxyl oxygen atom and Lys95 forming a salt bridge with Glu85.

The unique effect of M1 on mNT/NAF-1 [2Fe–2S] clusters led us to investigate the binding-mode of M1 to these NEET proteins. M1-mNT structure determined by X-ray crystallography at 1.65 Å resolution (Supplementary Table 1) shows the presence of an extra density that correspond to M1 molecule on each mNT monomer; monomer A and monomer B (Fig. 5 and Supplementary Figs. 4 and 5, respectively). In the M1-NAF-1 structure, determined at 1.74 Å resolution (Supplemental Table 1), only one extra density of M1 molecule is present in between two NAF-1 homodimers (Fig. 5 and Supplementary Figs. 4 and 5). According to the M1-mNT structure (Fig. 5a), M1 is in close proximity to the [2Fe–2S] cluster binding domain where the tetralin ring of M1 is oriented toward the [2Fe–2S] cluster while the distal phenyl ring is facing the *β*-cap domain. The M1 carboxyl group forms a salt bridge with Lys55 (2.2 Å distance between them) but it does not interact with His87 (3.5 and 3.7 Å distance). M1 is also oriented towards the side chain of Lys68 (*β*-cap domain of mNT) and forms a hydrogen bond with it (2.9 Å distance; Fig. 5a). The M1 molecule is produced as a racemic mixture.

The racemic mixture was tested in vivo (described below). There is a less than 10× difference in potency between the enantiomeric forms and no evidence of different pharmacokinetic behavior between the species, thereby all studies were performed with the mixture. The M1 molecule binds NAF-1 in the same area as observed in the mNT co-structure (Fig. 5b and Supplementary Fig. 6) with carbonyl oxygens of the thiophene core forming salt bridges with Lys81 (equivalent of Lys55 of mNT) and does not interact with NAF-1 His114 (equivalent of His87 of mNT). However, the structure of M1-NAF-1 indicates that M1 is unable to form a hydrogen bond with Lys95 (equivalent of Lys68 of mNT). Although mNT and NAF-1 share a high percentage of sequence and structure homology, NAF-1 possesses a glutamate residue at position 85 (not alanine as for mNT). Consequently, Lys95 is able to form a salt bridge with Glu85 (2.7 Å distance; Fig. 5b and Supplementary Fig. 5). The second binding poses observed on monomer B of mNT and NAF-1 monomer B, described in Supplementary Fig. 5, are probably due to the crystals packing effects shown before to affect ligand bindings to proteins^38,39^. Moreover, the surface of mNT and NAF-1 show hydrophobic residues facing the hydrophobic moieties of M1 (<4.5 Å) suggesting hydrophobic interactions between M1 and the mNT/NAF-1 protein (Supplementary Fig. 7).

## Discussion

Structure analysis of M1, that enhances the lability of mNT and NAF-1 [2Fe–2S] clusters (Fig. 1), identified the M1-mNT/NAF-1 molecular binding mode (Fig. 5). M1 binds strongly to the Lys55/Lys81 in mNT/NAF-1, respectively (Fig. 5 and Supplementary Fig. 4). Due to the considerable distance from His 87/His114 (mNT/NAF-1), M1 cannot bind to this amino acid which is a key mediator of proton facilitated cluster release^40^. In the native protein, hydrogen bond formation between Lys55/Lys81 and His87/His114 (mNT/NAF-1), is critical for cluster stability with His87/His114 in effect performing a gatekeeper function which is compromised upon M1 binding to Lys55/Lys81 to result in higher lability of the bound clusters. Failure of M1 to bind His87/His114 is an essential difference from the reported furosemide-mNT structure^41^ in which furosemide binds His87 (Supplementary Fig. 8) to stabilize binding of the [2Fe–2S] clusters to the mNT protein. A second important difference from the binding mode of furosemide to mNT is that the oxygen of the benzamide moiety of M1 molecule makes a hydrogen bond with mNT-Lys68 or shifts the position of NAF-1-Lys95 (Fig. 5 and Supplementary Fig. 4). Previous studies indicated that Lys68 mutagenesis (insertion at this position) destabilizes mNT’s [2Fe–2S] clusters^42^. Taken together the structures of M1-mNT/NAF-1 strongly suggest that in addition to His87/His114, interactions with Lys55/81 and cross-talk between the β-cap and the cluster binding area are also playing an important role in mNT and NAF-1 cluster lability/stability. This molecular model for pharmacological enhancement of [2Fe–2S] dissociation is supported by the binding features being preserved between mNT and NAF-1 (Fig. 5). For both proteins, M1 stimulates a significant acceleration of [2Fe–2S] clusters dissociation (Fig. 1).

Suppression of NEET protein expression has been shown in numerous cellular models to induce mLI accumulation resulting also in increased mROS^5,15,35,43–45^. Here we show that suppressed expression of mNT in INS-1E β-cells leads to increased mLI and mROS with the M1 molecule able to restore mLI and mROS to near normal levels and correct pathophysiological effects such as impaired insulin accumulation and secretion (Figs. 2 and 3). Similar pharmacological effects of M1 were also found in INS-1E β-cells with suppressed expression of NAF-1 (Figs. 2 and 3). These findings suggest that accelerated release of NEET proteins [2Fe–2S] clusters, a consequence of M1 binding, could overcome NEET protein deficiency by enhancing Fe/Fe–S export from inside the mitochondria by the remaining NEET proteins and/or their homologs. By contrast, under healthy conditions, the direct environment of NEET proteins is reduced^46^, and we hypothesize that the M1 molecule is not able to enhance the lability of the highly stable reduced [2Fe-2S] clusters of NEET proteins^24,47^

The normal function of β-pancreatic cells is to synthesize and secrete appropriate amounts of insulin in response to the prevailing circulating levels of glucose. Of direct relevance to the data presented here is that insulin gene expression and secretion are known to be sensitive to oxidative stress changes in pancreatic cells^48^. Furthermore, oxidative stress is known to disrupt insulin sensitivity in type 2 diabetes and insulin production in type 1 diabetes^33,37,48–50^. To extend our investigation of the role of M1 and NEET proteins in glucose homeostasis, we investigated the effects of M1 on glycemic parameters in *db/db* mice. We show that treating *db/db* mice with M1 significantly slowed blood glucose increase over the course of the study, improved blood glucose response to a 4 h fast, and significantly reduced hepatic lipids, without altering the rate of weight gain (Fig. 4).

In summary, our study expands from an atomic resolution understanding of the mode of action of M1 binding to the NEET proteins mNT and NAF-1, to observing corrective action on the cellular disorders induced by NEET protein deficiency in INS-1E β-cells and beyond finally to demonstrate the efficacy of M1 in correcting defective blood glucose homeostasis in a mouse model of diabetes. Taken together, the results presented propose that enhancing the lability of NEET proteins [2Fe–2S] clusters is an attractive, novel therapeutic mode of action and that M1 or its analogs could serve as candidates for a novel class of anti-diabetic drug.

## Methods

### Protein expression and purification

cDNAs encoding the soluble parts mNT (residue 33-108) or NAF-1 (residue 57–135) were inserted into the expression vector pet-28a+ (Novagen). mNT and NAF-1 proteins were expressed in *Escherichia coli* BL21-RIL grown in LB supplemented with 30 μg/mL kanamycin and 34 μg/mL chloramphenicol. At an OD600 of 0.6, the cells were supplemented with 0.75 mM FeCl_3,_ and the expression was activated using 0.25 mM of IPTG. Cell growth proceeded for an additional 12 h at 37 °C. Cells were then pelleted, resuspended in lysis buffer (20 mM Tris-HCl pH 8.0, 500 mM NaCl and 10 mM MgCl_2_. 3–5 mg of DNAse, and 3–5 mg of lysozymes added together with proteases inhibitor solution containing 200 mM aminocaproic acid, 200 mM benzamidine, and 200 mM PMSF) and disrupted with Microfluidizer^®^ cell disruptors. mNT or NAF-1 proteins were purified using Ni-agarose and size exclusion chromatography as described in refs. ^10,12^.

### M1 molecule synthesis

Molecule M1 was synthesized according to the method described in ref. ^51^.

### M1-mNT/NAF-1 *K*_d_ determination

The *K*_d_ of M1 binding to mNT/NAF-1 was determined using the ITC method with the MICROCAL PEAQ-ITC, Malvern instrument. Analyses were performed with MicroCal PEAQ-ITC Analysis Software. All experiments were performed by injecting 3 μL of M1 molecule at 500 µM in 100 mM Tris-HCl pH 8.0, 100 NaCl and 5% DMSO, into a 200 μL sample cell containing 50 µM mNT or NAF-1 solubilized in the same buffer (without DMSO) at 10 °C. Thirteen injections were performed with a spacing of 150S with a reference power of 10 μcal/s. A control experiment was performed by titration of the M1 molecule into buffer. The ITC measurements were fitted to a one-site binding model. *K*_d_ (±SD) value is the average of three independent repeats.

### Cluster stability assay

The stability of the mNT or NAF-1 [2Fe–2S] clusters were measured using the previously described stability-assay^52^. Briefly, the stability kinetics of the [2Fe–2S] cluster of mNT or NAF-1 was monitored by measuring the specific absorption peak the NEET protein [2Fe–2S] clusters at 458 nm using a Synergy™ H1 plate reader equipped with a temperature control apparatus set to 37 °C. The effect of M1 molecule was measured by incubating mNT or NAF-1 protein with M1 (1:3 molar ratio). Each curve represents the mean (±SD) of three experimental repeats.

### Co-crystallography and structure determination and refinement

The purified mNT/NAF-1 protein was dialyzed in a buffer containing 100 mM Tris-HCl (pH 8.0) and 100 mM NaCl. 20 mg of dialyzed mNT was then co-crystallized with the M1 molecule (3.3 mM) in 5% DMSO, using sitting drop vapor diffusion against a solution of 32% PEG-3000, 100 mM Tris-HCl (pH 8.0) and 100 mM NaCl. Small red crystals appeared after 48 h incubation at 20 °C. Prior to data collection, these crystals were cryoprotected in a solution containing 20% glycerol in the reservoir solution and were immediately flash cooled in liquid nitrogen. Crystallographic X-ray diffraction data of the crystals was collected at the BL14.2 beamline at BessyII, Berlin, Germany for mNT and at the ID-30A beamline at ESRF, Grenoble, at a temperature of 100 K and wavelength of 0.9184 Å/0.965. Crystals diffracted to the maximal resolution of 1.65 Å (mNT) and 1.74 Å (NAF-1), and data were integrated and scaled using XDS. The crystal belonged to the orthorhombic P212121 space group, with unit cell parameters *a* = 45.152 Å, *b* = 49.962 Å, *c* = 58.945 Å with two molecules in the asymmetric unit for mNT and *a* = 43.573 Å, *b* = 47.589 Å, c = 125.948 Å with two dimers in the asymmetric unit for NAF-1. The structure was solved by molecular replacement methods using Molrep^53^ at the resolution range of 35.0–4.5 Å using the atomic coordinates of a model derived from mitoNEET (PDB code: 3EW0) or from NAF-1 (PDB code: 3FMV), following removal of the N-termini up to Met44 and removal of all solvent molecules. The structure was then refined by seven cycles of restrained refinement using Refmac5 in the ccp4i suit^54^. The structure was fitted into electron density maps using the graphics program Coot^55^ and ligand fitted into the extra density using Coot. The structure was further refined using Refmac5^56^ restrained refinement with the maximum likelihood option.

### INS-1E cell growth

INS-1E *β*-pancreas cells were grown in 37 °C and 5% CO_2_ as previously described^37^, with RPMI 1640 and 11.1 mM D-glucose supplemented with 10% heat-inactivated fetal bovine serum, 100 U/ml penicillin, 100 µg/mL streptomycin, 10 mM HEPES, 2 mM L-glutamine, 1 mM sodium pyruvate, and 50 µM β-mercaptoethanol. The plasmid used to suppress mNT or NAF-1 protein expression [shRNA]^9^ was the pGFP-RS vector (purchased from OriGene). GenJuice (EMD Millipore) was used for transfection of INS-1E cells^43^. Cells transfected with shRNA vectors were treated with puromycin antibiotic (2 μg/mL) and stably transfected cell lines obtained by FACS sorting. Individual cell lines obtained were characterized for protein expression levels by western blot analysis^5,35,57^.

### Western blot analysis

Protein content was determined in extracts from control and transfected cell lines as previously described^37^. The Pierce 660 nm Protein Assay (catalog number 1861426), Ionic Detergent Compatibility Reagent (IDCR) (catalog number 22663), were used for protein quantification. Following SDS-PAGE separation of equal amounts of cellular proteins, proteins were transferred to nitrocellulose blots and incubated with antibodies against mNT/NAF-1 and *β*-actin (Abcam, MA). Peroxidase-conjugated Affinity Pure goat anti-rabbit and anti-mouse IgG from Jackson ImmunoResearch Laboratories (West Grove, PA) were used as secondary antibodies^5,35,37^.

### Epi-fluorescent microscopy analysis

INS-1E control and shRNA-mNT or shRNA-NAF-1 cells were plated and imaged for mLI accumulation using the probe RPA^5^. Mitochondrial ROS production and accumulation were determined by imaging cells with mito-SOX^TM^ Red (Invitrogen,182M36008). Mito-SOX^TM^ concentration was optimized for each cell line, on average for all INS-1E cell lines, a concentration of 2.5 μM Mito-SOX^TM^ was used. All fluorescence images were collected using an Epi-fluorescent microscope with a confocal (quality equivalent) opti-grid device (Nikon TE 2000 microscope equipped with a thermostatic stage and a Hamamatsu Orca-Era CCD camera) and driven by the Volocity 4 operating system (Improvision). The images collected were analyzed by velocity or image-J software programs.

### Insulin content and secretion

Insulin secretion of shNAF-1 and control INS-1E *β*-cells was evaluated by static incubation. Cells were pre-incubated for 30 min in RPMI 1640 containing 1.7 mM glucose and then consecutively incubated at 1.7 mM and 16.7 mM glucose for 1 h at 37 °C in 1 mL modified Krebs-Ringer bicarbonate buffer containing 20 mM HEPES and 0.25% BSA (KRBH-BSA). Medium was collected at the end of the basal (1.7 mM glucose) and stimulatory (16.7 mM glucose) incubations and centrifuged. Supernatants were then frozen at –20 °C pending insulin assay. Cell pellets resulting from centrifugation were subjected to repeated freeze-thaw cycles in 1.5 mL microfuge tubes containing 0.1% BSA in GB/NP-40 solution. Insulin immunoreactivity in the extracts and medium was determined using rat insulin ELISA Kit (Mercodia, Uppsala, Sweden)^37^.

### M1 molecule

M1 molecule was prepared in DMSO with a stock concentration of 10 mM. For INS-1E cell studies, M1 was diluted to 40 μM in RPMI 1640 with 11.1 mM D-glucose growth medium (see above) and M1 incubated with different INS-1E cell lines (final concentration, 5 µM) for 30 min. After incubation, M1 containing medium was exchanged for fresh growth medium, and cells were cultured for a further 30 min before they were imaged for mLI and mROS accumulation. For Insulin experiments, the M1 molecule (5 µM) was incubated with the different INS-1E cell lines for 3 h before analysis.

### Animal studies

All animal experiments were conducted in accordance with internationally accepted principles for the care and use of laboratory animals. Six-week-old male black BKS(D)-Leprdb/JOrlRj mice (*db*/*db*) obtained from Taconic (Denmark), entered the study at week-2 and were fed Purina 5008 chow (LabDiet) ad libitum for the duration of the study. On day-3, baseline measurements of body weight, fed blood glucose (fed BG) and glycated hemoglobin (HbA1c) were taken. Animals were randomized into treatment groups (10 mice each) by baseline HbA1c and fed BG. From day 0, treatment groups were administered vehicle (CMC (1.5% W/V), Tween 80 (0.25% V/V) in water, PO, QD), 3 mg/kg rosiglitazone (PO, QD), 7 mg/kg M1 molecule (PO, BID) or 20 mg/kg M1 molecule (PO, BID). Body weight and food intake were measured daily and after 31 days of test article administration, glycemic parameters were measured at sacrifice.

In the in vivo study male mice were used since it is the current common practice in preclinical drug discovery, in order to avoid possible variability from the estrus cycle which repeats every 4 to 5 days in mice.

### Statistics and reproducibility

Statistical significance tests (Student’s *t* test) for protein expression, insulin measurements and analysis of epi-fluorescent microscope and TEM images were performed using GraphPad Prism 8.3.1 software. Results are presented as mean ± SD and include all measured data points, or bar and include all measured data points. Differences were statistically significant if the Student’s *t* test produced a probability value of less than 5% (*P < 0.05; **P < 0.01; ***P < 0.001).

### Reporting summary

Further information on research design is available in the Nature Research Reporting Summary linked to this article.

## Supplementary information

Supplementary information

Description of Additional Supplementary Files

Supplementary data 1

Reporting Summary

## Data Availability

The atomic coordinates and the structure factors of the mitoNEET-M1 complex have been deposit in the RCSB-PDB with accession codes 7P0O and 7P0P for mNT-M1 and NAF-1-M1 respectively. Uncropped Western blot images are provided in Supplementary Fig. 9. All relevant data including the numerical and statistical source data that underlie the graphs in figures are provided in Supplementary Data 1.
