# Supplementary material for: An anti-diabetic drug targets NEET (CISD) proteins through destabilization of their [2Fe-2S] clusters
Source: Commun Biol. 2022 May 10;5:437. doi: 10.1038/s42003-022-03393-x (PMC9090738; doi:10.1038/s42003-022-03393-x)

Supplementary information:

An anti-diabetic drug targets NEET (CISD) proteins through destabilization of their [2Fe-2S] clusters

Henri-Baptiste Marjault[1,2*], Ola Karmi[1,3*], Ke Zuo[1,2], Dorit Michaeli[1], Yael Eisenberg-Domovich[1], Giulia Rossetti[2,4,5], Benoit de Chassey[6], Jacky Vonderscher[6], Ioav Cabantchik[1], Paolo Carloni[2,4,5,7], Ron Mittler[3], Oded Livnah[1], Eric Meldrum[6], Rachel Nechushtai[1**]

1 The Alexander Silberman Institute of Life Science and The Wolfson Centre for Applied Structural Biology, Faculty of Science and Mathematics, The Edmond J. Safra Campus at Givat Ram, The Hebrew University of Jerusalem, Jerusalem, 91904, Israel

2 Department of Physics, RWTH Aachen University, 52074 Aachen, Germany

3 Department of Surgery, University of Missouri School of Medicine, and Interdisciplinary Plant Group, Christopher S. Bond Life Sciences Center, University of Missouri, 1201 Rollins St, Columbia, MO 65211, USA

4 Computational Biomedicine Section, Institute of Advanced Simulation IAS-5 and Institute of Neuroscience and Medicine INM-9, Forschungszentrum Jülich GmbH, 52425 Jülich, Germany

5 Computational Biomedicine, Institute of Advanced Simulation IAS-5 and Institute of Neuroscience and Medicine INM-9, For-schungszentrum Jülich GmbH, 52425 Jülich, Germany

6 ENYO-Pharma, Bioserra 1, 60 Avenue Rockefeller Bâtiment B, 69008 Lyon, France

7 JARA Institute: Molecular Neuroscience and Imaging, Institute of Neuroscience and Medicine INM-11, Forschungszentrum Jülich GmbH, 52425 Jülich, Germany

[*]Equally contributed to the experimental work presented

[**]To whom correspondence should be addressed: Rachel@mail.huji.ac.il

Supplementary Table 1. Data collection and refinement statistics of m1-mNT and M1-NAF-1 complexes

	mNT-M1	NAF-1-M1
**Data collection**		
Space group	P 2_1 2_1 2_1	P 2_1 2_1 2_1
Cell dimensions		
a, b, c (Å)	45.15, 49.96, 58.95	43.57, 47.59, 125.95
α, β, γ (°)	90 90 90	90 90 90
Resolution (Å)	45.15-1.65(1.68-1.65)	47.59-1.74 (1.77-1.74)
R_{merge}	0.087 (1.88)	0.127 (1.75)
I / σI	10.2 (1.0)	8.8 (0.9)
Completeness (%)	99.8 (100)	99.4 (100.0)
Redundancy	5.3 (5.6)	4.6 (4.8)
CC1/2	0.99 (0.56)	0.99 (0.38)
**Refinement**		
Resolution (Å)	45.15-1.65	41.21-1.74
No. reflections	16,607	26,499
R_{work} / R_{free}	17.4/ 23.6	20.16/25.08
No. atoms		
Protein	1063	2077
Ligand/ion	52/22	35/32
Water	50	79
B-factors		
Protein	37.12	33.70
Ligand/ion	C=70.51<br>D=43.62	35.11
Water	44.51	36.24
R.m.s. deviations		
Bond lengths (Å)	0.012	0.014
Bond angles (°)	2.19	2.17

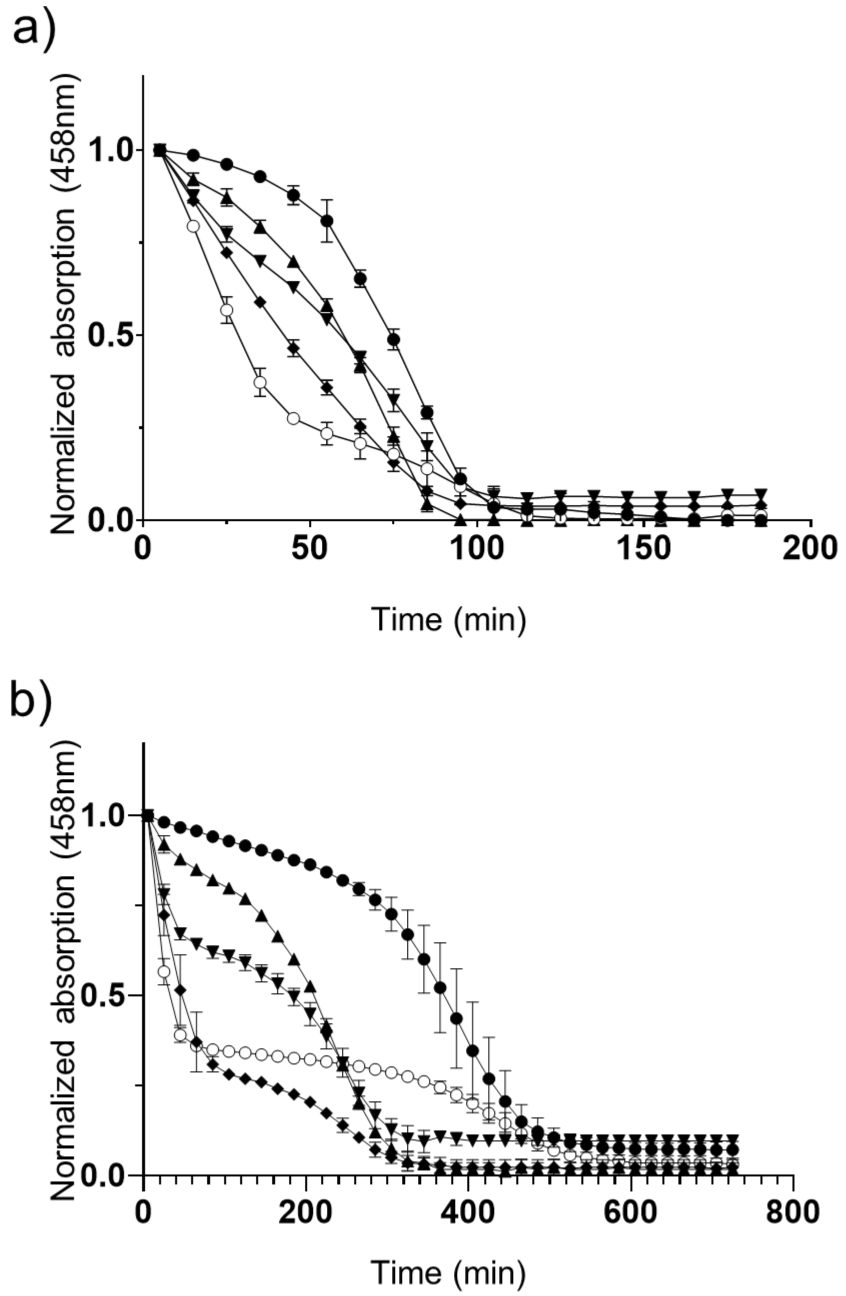

Supplementary Figure 1. The M1 molecule destabilizes the NEET proteins' [2Fe-2S] clusters in a dose-dependent manner. The [2Fe-2S] cluster release from 20 µM of mNT (a) and NAF-1 (b) proteins was monitored following the characteristic absorption peak of bound [2Fe–2S] at 458 nm. Several concentrations of M1 molecule (black triangle/10 µM; inverse black triangle/20 µM; black diamond/40 µM and empty circle/60 µM) were added to the NEET proteins and the effect of M1 additions was compared to the cluster stabilities of mNT and NAF-1 in the absence of the M1 molecule (filled circle). The temperature was 37 °C and pH 6.0; the error bar represents the standard deviation; n=3.

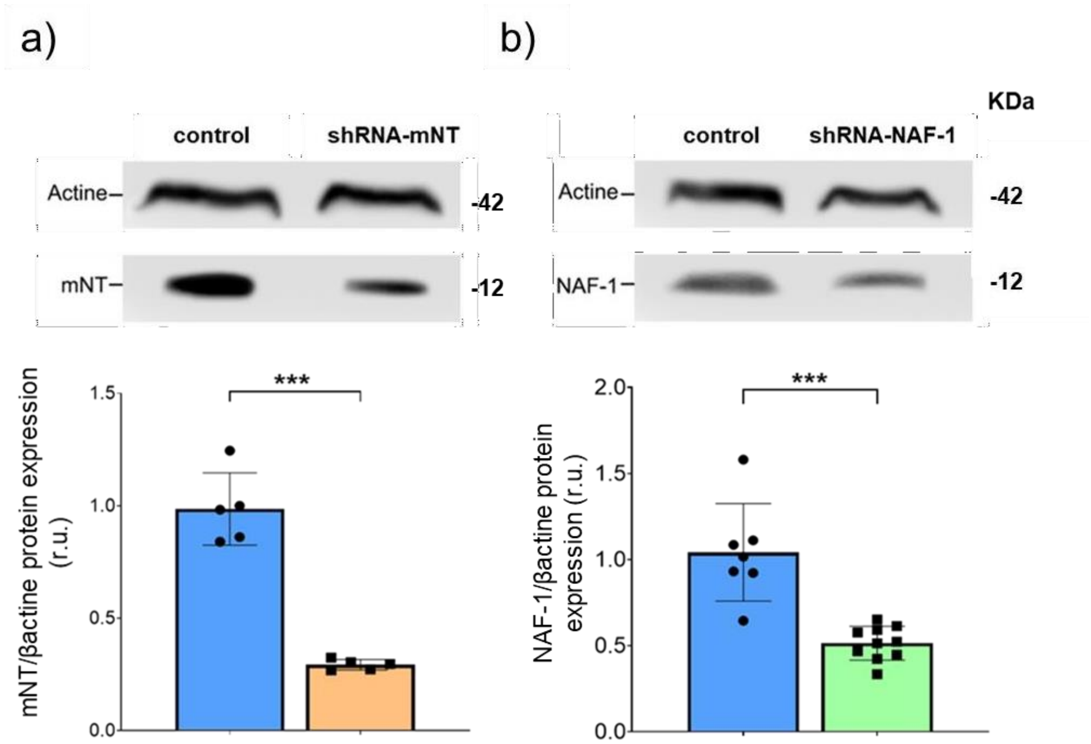

Supplementary Figure 2. Generation of the INS-1E cell line with suppressed mNT or NAF-1 protein expression levels. mNT or NAF-1 expression was down-regulated using shRNA against mNT or NAF-1 mRNA. **a&b**, levels of mNT and NAF-1 were determined by Western Blots (upper panel) and the level of protein in down regulated cells (shRNA-mNT, orange, n=5; shRNA-NAF-1, green, n=7) was normalized and compared to control (blue). The error bar represents the standard deviation ***, $p<0.001$.

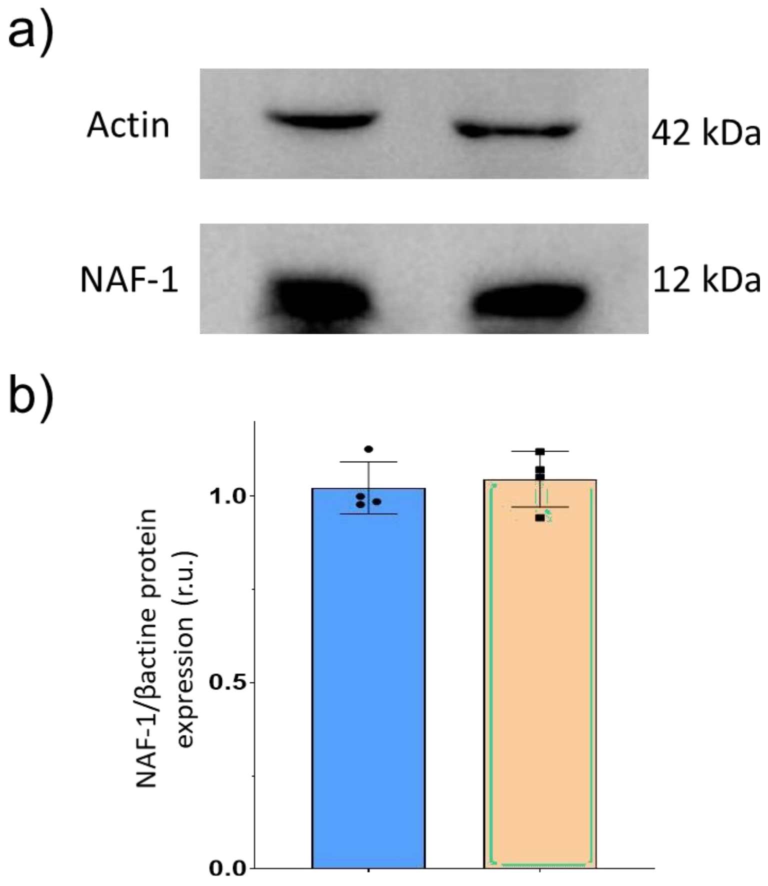

Supplementary Figure 3. NAF-1 protein expression levels in the INS-1E cells with downregulated mNT protein expression. mNT expression was down-regulated using shRNA against mNT and the levels of the NAF-1 protein were determined by Western Blot analysis (a). The levels of the NAF-1 protein in down regulated cells (shRNA-mNT, n=4 – b-orange bar) were normalized and compared to control INS-1E control cells transfected with the empty vector (b – blue bar). The error bar represents the standard deviation

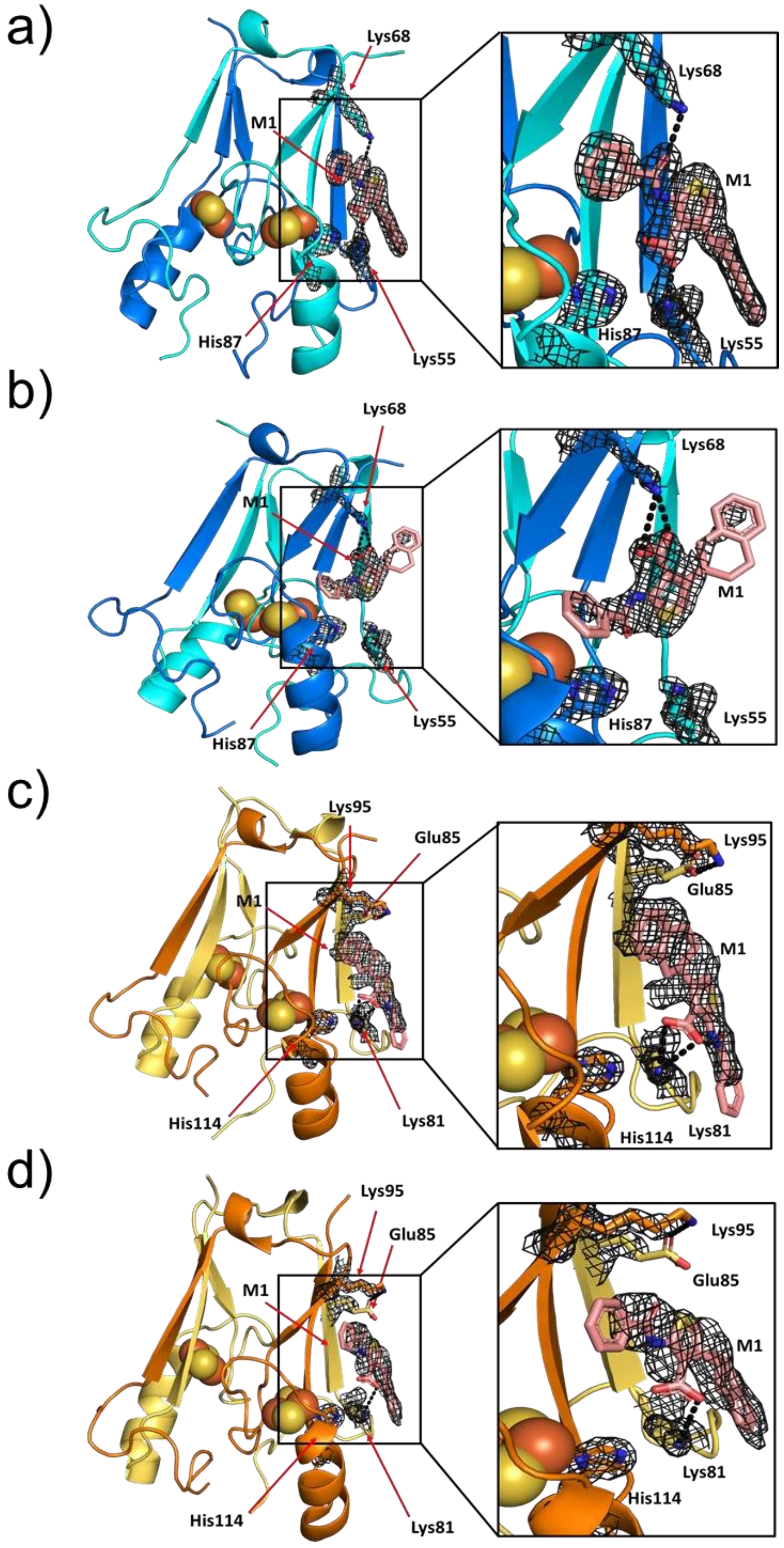

Supplementary Figure 4. Electron density maps of the M1 ligand bound to mNT and NAF-1 protein. 2Fo–Fc map contoured (grey mesh with σ1.5) for the ligand and the His 87, Lys 55 and 68 residues for mNT (**a** and **b**) or for residues His114, Lys 81, 95 and Glu85 for NAF-1 (**c** and **d)**.

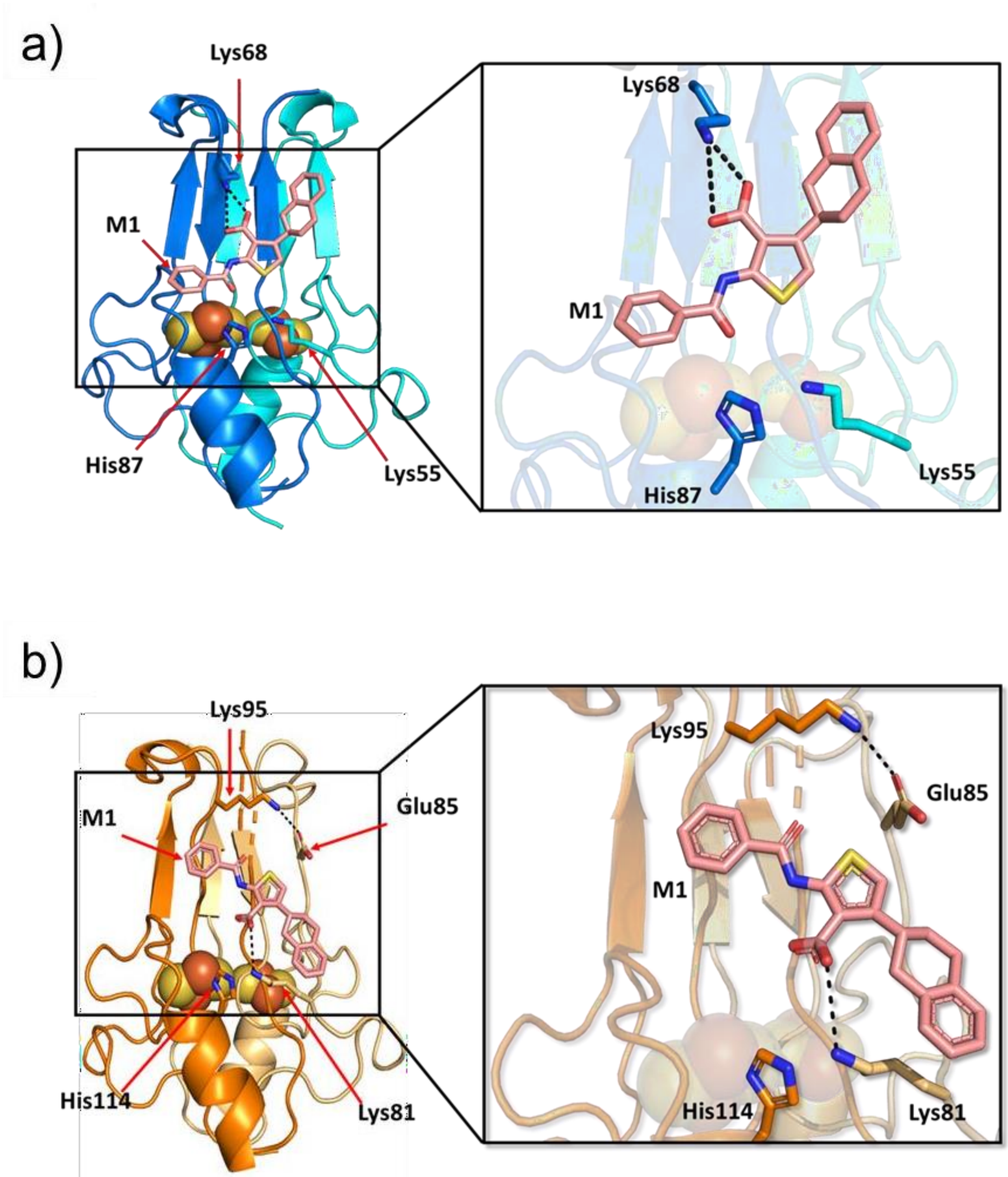

Supplementary Figure 5. Second binding poses of M1 molecule on mNT and NAF-1. **a.** A second binding pose of M1 molecule was observed on the second mNT's monomer – monomer B. It may very well be that the effects induced by the packing of the crystal induced a "flip" of the M1 molecule. Hence, M1 oxygens of the carboxylic acid function are oriented towards Lys68 and are predict to interact it. **b.** M1 molecule was found to be bound to one of the two NAF-1 homodimers (to its monomer B) at the interface between the two homodimers. The binding is approximately on the same area as in monomer A of NAF-1 (Fig. 5b) but in another orientation. In monomer B the M1 molecule is predicated to interact with only Lys 81. Additionally, Lys95 moves toward Glu-85 to form a salt bridge interaction.

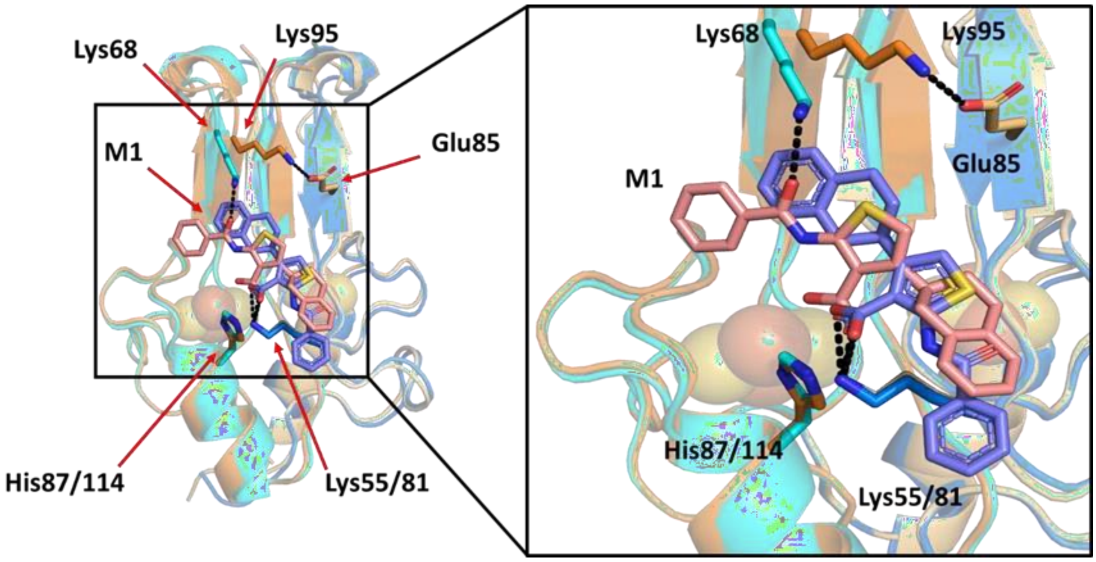

Supplementary Figure 6. Superimposition of the binding pose of M1 molecule with mNT and NAF-1 monomers A. mNT (cyan/blue marine color) and NAF-1 (orange/light orange) are superimposed to highlight the two different binding modes of M1 (pink for mNT and purple for NAF-1).

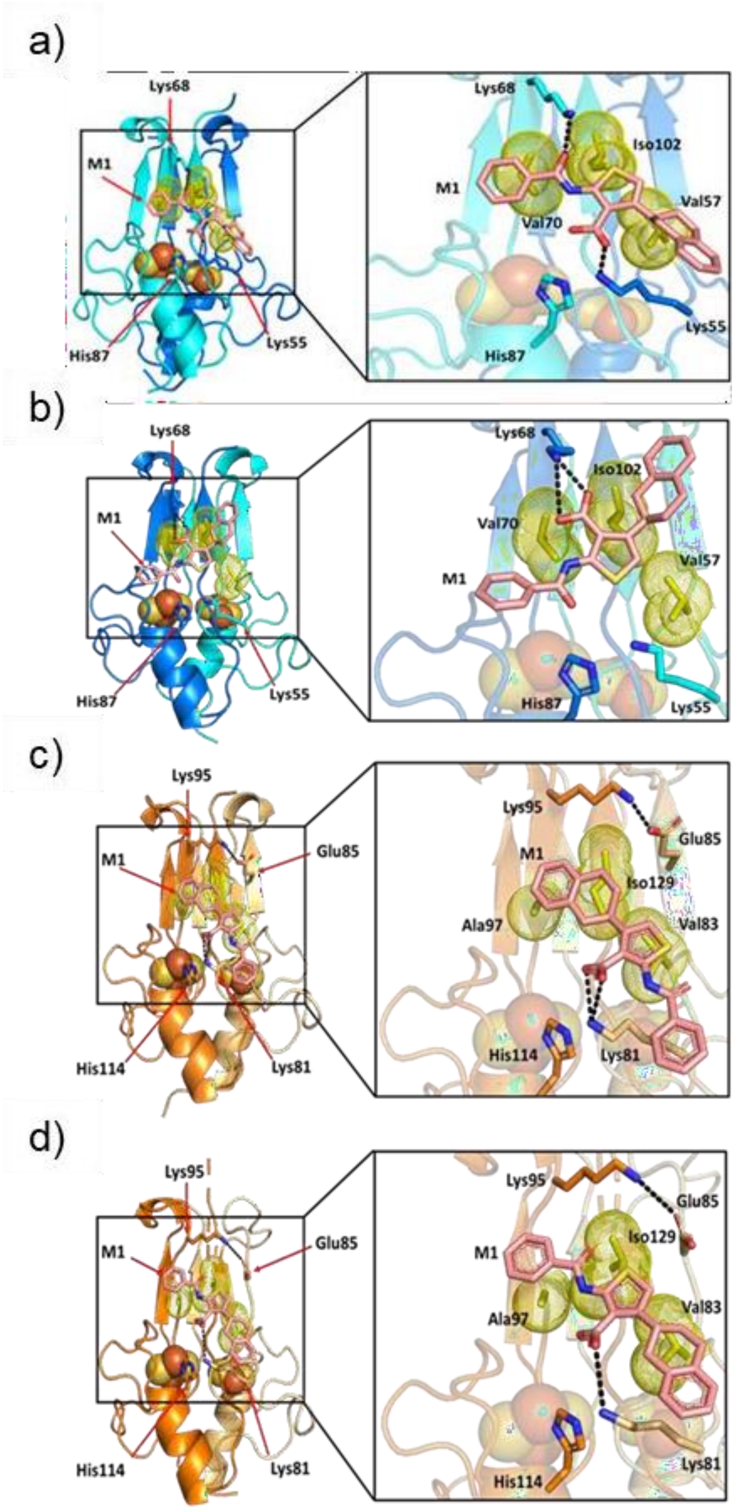

Supplementary Figure 7. Hydrophobic interactions between M1 and mNT or NAF-1. a&b. The binding surface of mNT protein (cyan & blue marine) where hydrophobic residues that can form hydrophobic interactions with M1's hydrophobic moieties are shown in yellow clouds. **c&d**; Similar hydrophobic binding surfaces are observed for NAF-1 protein (orange/light orange) with the same repartition of hydrophobic residue (yellow cloud) that could form hydrophobic interactions with M1. Both for mNT and NAF-1 the hydrophobic interactions are shown for monomer A(**a&c**) and monomer B (**c&d**).

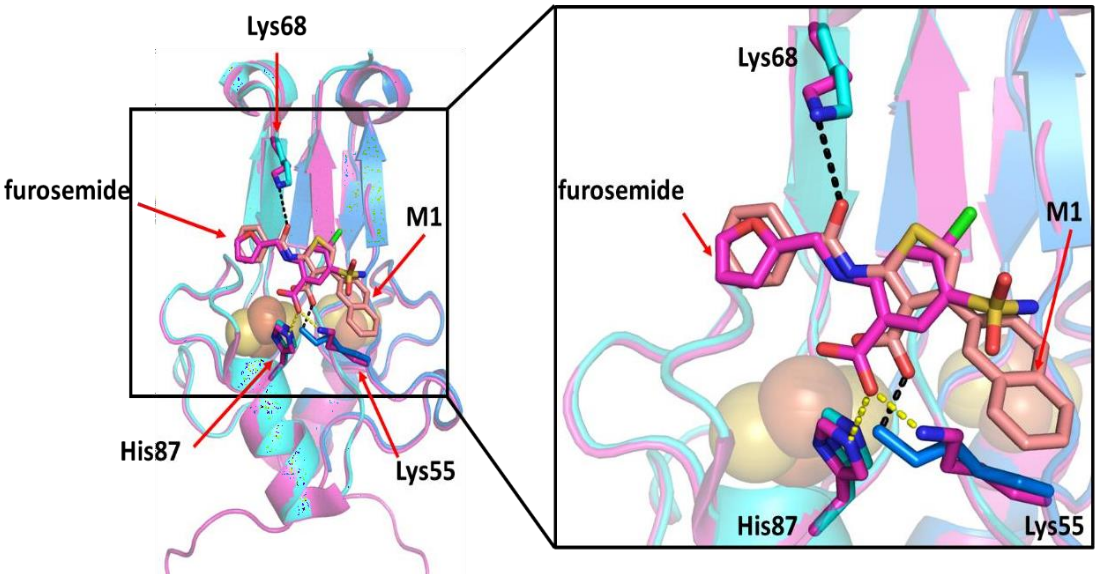

Supplementary Figure 8. Superimposition of mNT-M1 structure (cyan/blue) and mNT-furosemide binding (6DE9/purple). M1 (pink) molecule and furosemide (violet) are binding on the same area of mNT. M1 molecule is predicted to interact with Lys55 & Lys68 but *not* with His87. Furosemide interacts Lys55 and the His87. The interaction with His87 is predicated to convey furosemide its [2F-2S] stabilization properties.

Supplementary Figure 9:

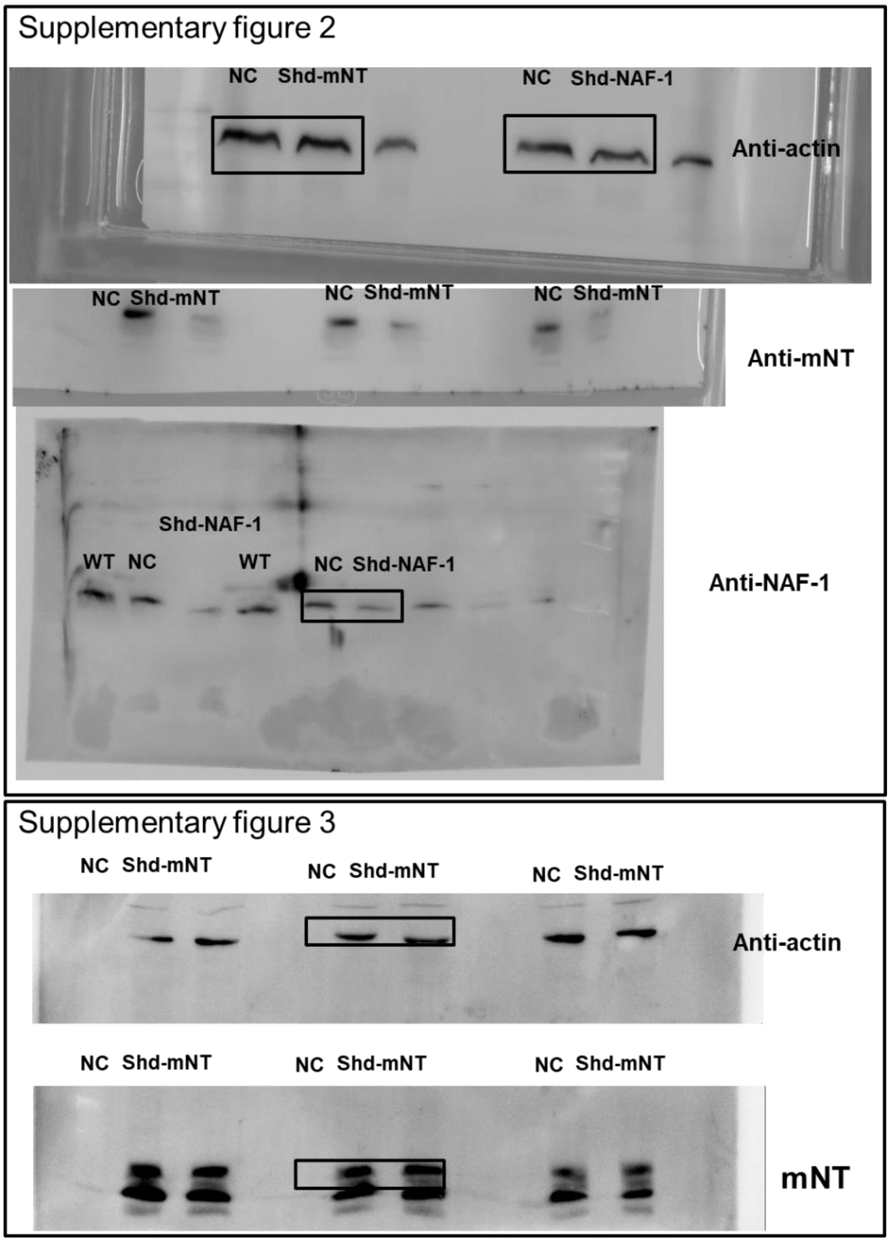

Supplement: Supplementary file 1 — Supplementary information [file 42003_2022_3393_MOESM1_ESM.pdf]
