# Supplementary material for: An anti-diabetic drug targets NEET (CISD) proteins through destabilization of their [2Fe-2S] clusters
Source: Commun Biol. 2022 May 10;5:437. doi: 10.1038/s42003-022-03393-x (PMC9090738; doi:10.1038/s42003-022-03393-x)
Supplement: Supplementary file 2 — Description of Additional Supplementary Files [file 42003_2022_3393_MOESM2_ESM.pdf]

Description of Additional Supplementary Files

File name: Supplementary Data 1

Description: Source data that underline the graphs in figures.
