# Supplementary material for: An anti-diabetic drug targets NEET (CISD) proteins through destabilization of their [2Fe-2S] clusters
Source: Commun Biol. 2022 May 10;5:437. doi: 10.1038/s42003-022-03393-x (PMC9090738; doi:10.1038/s42003-022-03393-x)
Supplement: Supplementary file 4 — Reporting Summary [file 42003_2022_3393_MOESM4_ESM.pdf]

nature portfolio

Corresponding author(s): DBPR-COMMSBIO-21-2647A

Last updated by author(s): Mar 16, 2022

Reporting Summary

Nature Portfolio wishes to improve the reproducibility of the work that we publish. This form provides structure for consistency and transparency in reporting. For further information on Nature Portfolio policies, see our Editorial Policies and the Editorial Policy Checklist.

Statistics

For all statistical analyses, confirm that the following items are present in the figure legend, table legend, main text, or Methods section.

n/a	Confirmed	
☐	☒	The exact sample size (*n*) for each experimental group/condition, given as a discrete number and unit of measurement
☐	☒	A statement on whether measurements were taken from distinct samples or whether the same sample was measured repeatedly
☐	☒	The statistical test(s) used AND whether they are one- or two-sided<br>*Only common tests should be described solely by name; describe more complex techniques in the Methods section.*
☐	☒	A description of all covariates tested
☐	☒	A description of any assumptions or corrections, such as tests of normality and adjustment for multiple comparisons
☐	☒	A full description of the statistical parameters including central tendency (e.g. means) or other basic estimates (e.g. regression coefficient) AND variation (e.g. standard deviation) or associated estimates of uncertainty (e.g. confidence intervals)
☐	☒	For null hypothesis testing, the test statistic (e.g. F, t, r) with confidence intervals, effect sizes, degrees of freedom and P value noted<br>*Give P values as exact values whenever suitable.*
☐	☒	For Bayesian analysis, information on the choice of priors and Markov chain Monte Carlo settings
☐	☒	For hierarchical and complex designs, identification of the appropriate level for tests and full reporting of outcomes
☐	☒	Estimates of effect sizes (e.g. Cohen's *d*, Pearson's *r*), indicating how they were calculated

Our web collection on statistics for biologists contains articles on many of the points above.

Software and code

Policy information about availability of computer code

Data collection	Crystallographic x-ray diffraction data of the crystals was collected at the BL14.2 beamline at BessyII, Berlin, Germany for mNT and at the ID-30A beamline at ESRF, Grenoble, at a temperature of 100 K and wavelength of 0.9184 Å/0.965.
Data analysis	Graphpad, imageJ, Coot, ccp4i suite(refmacc and Molprep)

For manuscripts utilizing custom algorithms or software that are central to the research but not yet described in published literature, software must be made available to editors and reviewers. We strongly encourage code deposition in a community repository (e.g. GitHub). See the Nature Portfolio guidelines for submitting code & software for further information.

Data

Policy information about availability of data

All manuscripts must include a data availability statement. This statement should provide the following information, where applicable:

- Accession codes, unique identifiers, or web links for publicly available datasets
- A description of any restrictions on data availability
- For clinical datasets or third party data, please ensure that the statement adheres to our policy

7P0O and 7POP, uncropped Western blot images are provided in Supplementary Figure 8. All relevant data including the numerical and statistical source data that underlie the graphs in figures are provided in Supplementary Data 1.

Field-specific reporting

Please select the one below that is the best fit for your research. If you are not sure, read the appropriate sections before making your selection.

☒ Life sciences ☐ Behavioural & social sciences ☐ Ecological, evolutionary & environmental sciences

For a reference copy of the document with all sections, see nature.com/documents/nr-reporting-summary-flat.pdf

Life sciences study design

All studies must disclose on these points even when the disclosure is negative.

Sample size	every experiment contain at least 3 sample per condition exept for the ITC mesurment (one sample a time)
Data exclusions	no data were exclude
Replication	all experiment were all replicate and are shown as dot on the figure results. A minimum of 3 replication per experiement were done.
Randomization	Biochemistry: all experiment with pure protein were done using on purification preparation per experiment<br>cell work: all cell used for each cell line were plate from the same population (per cell line) for each experiment<br>Mice work: Animals were randomized into treatment groups (10 mice each) by baseline HbA1c and fed BG
Blinding	no need of blinding for this study

Reporting for specific materials, systems and methods

We require information from authors about some types of materials, experimental systems and methods used in many studies. Here, indicate whether each material, system or method listed is relevant to your study. If you are not sure if a list item applies to your research, read the appropriate section before selecting a response.

Materials & experimental systems

n/a	Involved in the study	
☐	☒	Antibodies
☐	☒	Eukaryotic cell lines
☒	☐	Palaeontology and archaeology
☐	☒	Animals and other organisms
☒	☐	Human research participants
☒	☐	Clinical data
☒	☐	Dual use research of concern

Methods

n/a	Involved in the study	
☒	☐	ChIP-seq
☒	☐	Flow cytometry
☒	☐	MRI-based neuroimaging

Antibodies

Antibodies used	anti b-actine (1:1000, abcam) and anti-mNT (1:500) and NAF-1 (1:500) obtain from our in house pure protein and produce in rabbit.
Validation	Western Blot

Eukaryotic cell lines

Policy information about cell lines

Cell line source(s)	INS-1E (RRID:CVCL_0351)
Authentication	cell certificate
Mycoplasma contamination	All cell line were tested negatif for Mycoplasma contamination
Commonly misidentified lines<br>(See ICLAC register)	no applicable

Animals and other organisms

Policy information about studies involving animals; ARRIVE guidelines recommended for reporting animal research

Laboratory animals	Six-week-old male black BKS(D)-Leprdb/JOrlRj mice (db/db)
Wild animals	this study did not involve wild animals
Field-collected samples	The study did not involve samples from Field collection
Ethics oversight	Taconic (Denmark)

Note that full information on the approval of the study protocol must also be provided in the manuscript.
